# 3D Printed Integrated Gradient-Conductive MXene/CNT/Polyimide Aerogel Frames for Electromagnetic Interference Shielding with Ultra-Low Reflection

**DOI:** 10.1007/s40820-023-01017-5

**Published:** 2023-02-08

**Authors:** Tiantian Xue, Yi Yang, Dingyi Yu, Qamar Wali, Zhenyu Wang, Xuesong Cao, Wei Fan, Tianxi Liu

**Affiliations:** 1 State Key Laboratory for Modification of Chemical Fibers and Polymer Materials, College of Materials Science and Engineering, Donghua University, 2999 North Renmin Road, Shanghai, 201620 People’s Republic of China; 2 Key Laboratory of Synthetic and Biological Colloids, Ministry of Education, School of Chemical and Material Engineering, Jiangnan University, Wuxi, 214122 People’s Republic of China; 3 Institute of Environmental Processes and Pollution Control, School of Environment and Civil Engineering, Jiangnan University, Wuxi, 214122 People’s Republic of China; 4 NUTECH School of Applied Sciences & Humanities, National University of Technology, Islamabad, 44000 Pakistan

**Keywords:** 3D printing, MXene/CNT/Polyimide aerogel, Gradient-conductive, Electromagnetic interference shielding

## Abstract

Construction of advanced electromagnetic interference (EMI) shielding materials with miniaturized, programmable structure and low reflection are promising but challenging. Herein, an integrated transition-metal carbides/carbon nanotube/polyimide (gradient-conductive MXene/CNT/PI, GCMCP) aerogel frame with hierarchical porous structure and gradient-conductivity has been constructed to achieve EMI shielding with ultra-low reflection. The gradient-conductive structures are obtained by continuous 3D printing of MXene/CNT/poly (amic acid) inks with different CNT contents, where the slightly conductive top layer serves as EM absorption layer and the highly conductive bottom layer as reflection layer. In addition, the hierarchical porous structure could extend the EM dissipation path and dissipate EM by multiple reflections. Consequently, the GCMCP aerogel frames exhibit an excellent average EMI shielding efficiency (68.2 dB) and low reflection (*R* = 0.23). Furthermore, the GCMCP aerogel frames with miniaturized and programmable structures can be used as EMI shielding gaskets and effectively block wireless power transmission, which shows a prosperous application prospect in defense industry and aerospace.

**Supplementary Information:**

The online version contains supplementary material available at 10.1007/s40820-023-01017-5.

## Highlights

- The MXene/CNT/Polyimide aerogel frame with integrated gradient-conductive structure was constructed by MXene/CNT/poly(amic acid) composite inks with different CNT contents via 3D printing technology.
- The integrated gradient-conductivity and hierarchical porous structure of MXene/CNT/Polyimide aerogel frame rendered excellent electromagnetic interference shielding performance and ultra low reflection.

**Supplementary Information:**

The online version contains supplementary material available at 10.1007/s40820-023-01017-5.

## Graphical abstract

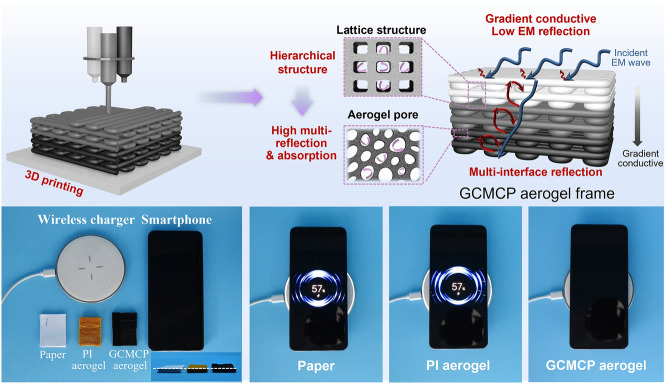

## Introduction

With the prosperity of microelectronics and 5th generation mobile communication technology, it is necessary to develop lightweight, programmable structure and ultra-efficient electromagnetic interference (EMI) shielding materials to ensure proper operation of electronic devices and human health [1–3]. Currently, polymer-based conductive composites are rapidly developed as EMI shielding materials benefiting to their corrosion resistance, easy processing and flexible design of conductive networks [4–6]. However, polymer-based EMI shielding materials with a homogeneous high conductive network exhibit high reflection of electromagnetic waves into free space, leading to serious secondary electromagnetic radiation contamination [7–9]. To minimize the secondary electromagnetic radiation pollution, absorption-dominated EMI shielding polymer composites with low reflection are greatly desired for next-generation electronic devices. Yet, polymer-based EMI shielding materials with low conductivity would effectively reduce the reflection of electromagnetic waves, but inevitably reduces the EMI shielding efficiency (EMI *SE*) due to weak absorption losses within the material [10, 11]. Therefore, an improved understanding between structure and functionality of polymer-based EMI shielding material is critical for design and development of advanced EMI shielding materials with low reflection and high absorption.

In recent years, lightweight aerogels with high porosity are considered as promising materials for EMI shielding [4, 12]. Benefit to the three-dimensional (3D) porous structure of aerogel, the incident electromagnetic waves can be multiple reflected/scattered and lost in the porous structure, thus enhancing the EMI shielding performance, especially its electromagnetic wave absorption ability [13, 14]. For instance, Zhao et al. [15] reported transition-metal carbides (MXenes)/graphene oxide (GO) hybrid aerogel with aligned cellular microstructure and high conductivity (1085 S m^−1^), which effectively performs electron transfer and microwave attenuation exhibiting high EMI shielding effectiveness (EMI *SE* = 50 dB in the X band). In addition, Zeng et al. [16] prepared silver nanowire/cellulose composite aerogels with laminar, honeycomb and random porous structures by adjusting the freezing method, where the laminar-structured aerogels have high EMI shielding properties and low density. However, this uniform conductive network of aerogels leads to an impedance mismatch between the air-material interface, causing electromagnetic waves to be reflected into free space and forming secondary electromagnetic radiation pollution. Therefore, Xu et al. [17] constructed a gradient-conductive structure via sedimentation of the conductive filler with different densities, which can absorb-reflect-reabsorb electromagnetic wave, showing an excellent EMI SE value of nearly 87.2 dB and a reflection coefficient (*R*) of 0.39. Hu et al. [18] constructed a low-conductive impedance matching layer on top of the highly conductive material by a layer-by-layer method, thus reducing the reflection of electromagnetic waves and obtaining low *SE*_R_ of 0.29 dB. Therefore, constructing a porous structure as well as reasonable gradient-conductive structure is a feasible way to effectively enhance the EMI *SE* and reduce reflection coefficient [19–21]. However, due to the cumbersome and unreliable construction of gradient-conductive structures via sedimentation or layer-by-layer methods, it is still a challenge to fabricate absorption-dominated polymer-based EMI shielding materials with low reflection coefficient.

3D printing technology is a promising technology with the advantages of complex structure formation and multi-component integration [22–27]. The integrated construction of gradient-conductive structure by 3D printing technology is expected to be a new strategy for integrated and customized preparation of polymer-based electromagnetic shielding materials with low reflection and high absorption. Herein, we report an effective strategy to fabricate gradient-conductive transition-metal carbides/carbon nanotube/polyimide (gradient-conductive MXene/CNT/PI, GCMCP) aerogel frames with hierarchical porous structure via 3D printing technology. The integrated gradient-conductive structure from bottom to top is directly formed by continuous 3D printing of MXene/CNT/poly (amic acid) (MXene/CNT/PAA) composite inks with different CNT contents. In the GCMCP aerogel frames, the slightly conductive top layer of MCP aerogel serves as the EM absorption layer (impedance matching layer), while the highly conductive bottom layer of MCP aerogel as an EM reflection layer, forming an absorption-reflection-reabsorption interface. In addition, the hierarchical porous structure (lattice macrostructure and aerogel porous microstructure) of GCMCP aerogel frames extends the electromagnetic wave dissipation path by limiting the incident waves to enter the material in a specific direction and dissipates the electromagnetic wave by multiple reflections. As expected, the GCMCP aerogel frames shows high EMI *SE* (68.2 dB) as well as low reflection coefficient (*R* = 0.23). The custom-designed GCMCP aerogel frames as electromagnetic shielding gasket is further demonstrated by a practical application in blocking power transmission. This work provides a novel strategy for designing EMI shielding materials with low EM wave reflection, which has a prosperous application potential in the high-end EMI shielding fields, such as the national defense industry and aerospace.

## Experimental Section

### Materials

Lithium fluoride (LiF, 99%) was purchased by Sigma-Aldrich Co., Ltd. Ti_3_AlC_2_ powder was obtained from Jilin 11 Technology Co., Ltd. 4,4’-diaminodiphenyl ether (ODA, 99%), pyromellitic dianhydride (PMDA), N, N-dimethylacetamide (DMAc), hydrochloric acid (HCl, 37%), and triethylamine (TEA) were all purchased from Sinopharm Chemical Reagent Co. Multiwalled carbon nanotubes (CNT, TNWDM-M8) were acquired from Chengdu Organic Chemicals Co. Ltd.

### Preparation of MXene Sheets

MXene sheets with accordion-like structure were successfully synthesized through etching Ti_3_AlC_2_ powder in the LiF/HCl solution, as noted in reported work [28, 29]. Firstly, LiF (1 g) was added into HCl (20 mL, 9 mol L^−1^) and stirred (500 r min^−1^) at room temperature for 10 min. Subsequently, Ti_3_AlC_2_ powder was added into the above solution, and the mixed solution was continuously stirred for 24 h at 35 ℃. The multilayered MXene was washed with deionized water for 7–8 times, and centrifuged at 5,000 rpm for 5 min. Finally, the multilayer MXene dispersion was subjected to high-speed centrifugation at 10,000 rpm for 60 min and then freeze-drying to obtain the few-layered MXene powder.

### Preparation of the MXene/CNT/PAA Composite Inks

The poly (amic acid) (PAA) salt precursors are prepared based on previous work [30–32]. 0.5 g PAA was first dispersed in 10 mL MXene suspension (50 mg mL^−1^) and stirred for 6 h to obtain MXene/CNT/PAA-0 composite ink. Then, the CNT (0.25 g, 25 mg mL^−1^) was added to the above MXene/PAA ink to obtain MXene/CNT/PAA-25 composite ink. Different conductive inks were obtained by changing CNT content. According to the different CNT contents (0, 25, 50 and 100 mg mL^−1^), the composite inks were named MXene/CNT/PAA-0, MXene/CNT/PAA-25, MXene/CNT/PAA-50 and MXene/CNT/PAA-100, respectively (Table S1).

### 3D Printing Process and Fabrication of Gradient-Conductive MXene/CNT/Polyimide Aerogel Frames

The prepared MXene/CNT/PAA composite inks with different CNT content composite loaded into the separate syringes and extrusion-printed using a 3D printer (BP6601, China). Here, we designed geometric pattern with varies shapes by CAD software in advance, and then imported them into the printer. Firstly, we used the MXene/CNT/PAA-100 composite ink to print lattice structure as the bottom layer, then switch to MXene/CNT/PAA-25 composite ink to print lattice structure as the middle layer, and the top-layered lattice structure is printed with MXene/CNT/PAA-0 composite ink. Finally, the GCMCP-(0–25–100) aerogel frame could be obtained by freeze-drying for removing the ice crystal and thermal imidization at 300 °C in an argon atmosphere. We constructed three gradient-conductive structure, named GCMCP-(X_1_-X_2_-X_3_), in which X_1_, X_2_ X_3_ represent the CNT content in the top layer, middle layer and bottom layer of GCMCP aerogel frame, respectively (Table S2).

### Characterizations

The morphologies of the GCMCP aerogel frame and multilayered MXene were investigated by scanning electron microscope (FESEM, JSM-7500F, Japan). The few-layered MXene sheets were characterized by transmission electron microscopy (TEM, JEM-2100F, Japan). The XRD spectra of MAX(Ti_3_AlC_2_) and MXene were characterized by X-ray diffraction (DX-2700BH, China). Fourier-transform infrared spectra (FTIR) were recorded with a Nicolet 6700 FTIR spectrophotometer (Bruker Spectrum Instruments, USA). The rheological properties of the MXene/CNT/PAA composite inks were measured by modular compact rheometer (MCR302, China), and the scanning range of shear rate was of 0.01–100 s^−1^. The mechanical properties were measured in the electronic universal testing machine (UTM2102, China) with a sensor of 100 N. The electrical conductivity was recorded by four-probe tester (MCP-T610, Mitsubishi Chemical). Conductivity of the gradient structure is calculated according to Eq. (1) [33]:

1 $$ \sigma = \frac{l}{{Rw{\text{d}}}} $$

where $\sigma$ is the conductivity; *R* is the resistance of sample; $l$, w and d are the thickness, width and distance between test electrodes, respectively. The resistance was measured by the resistance tester (2612B, Keithley, USA). The EMI shielding characteristics of aerogels were evaluated by the vector network analyzer (ZNB40, China) in X-band frequency range (8.2–12.4 GHz). The EMI shielding parameters are as follows: scattering parameters (*S*_11_ and *S*_21_), reflection coefficient (*R*), transmission coefficient (*T*), absorption coefficient (*A*), total EMI shielding effectiveness (*SE*_T_), electromagnetic waves reflection (*SE*_R_), electromagnetic waves absorption (*SE*_A_), and the calculation formula is as follows [15, 19]:

2 $$ R = \left| {S_{11} } \right|^{2} $$

3 $$ T = \left| {S_{21} } \right|^{2} $$

4 $$ A = 1 - \left( {R + T} \right) $$

5 $$ SE_{T} = 10 \log \left( \frac{1}{T} \right) $$

6 $$ SE_{R} = 10 \log \left( {\frac{1}{1 - R}} \right) $$

7 $$ SE_{A} = 10 \log \left( {\frac{1 - R}{T}} \right) $$

## Results and Discussion

### Fabrication of the GCMCP Aerogel Frames

**Fig. 1:**
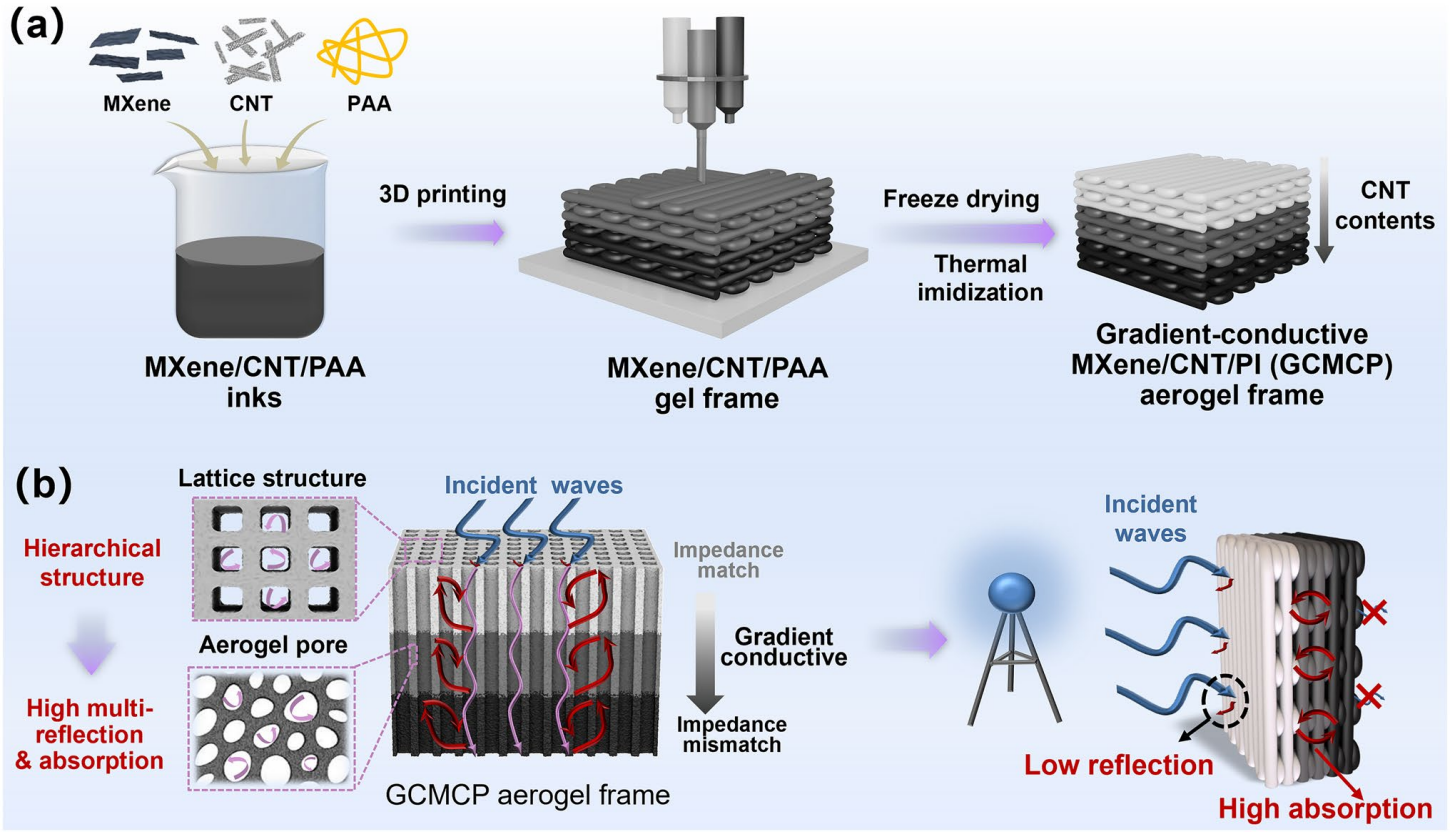
(**a**) Schematic illustration of the fabrication process of the GCMCP aerogel frame. (**b**) Schematic diagram of EMI shielding mechanism of GCMCP aerogel frame and its application in the EMI shielding to reduce electromagnetic radiation pollution

The fabrication process of 3D printed gradient-conductive MXene/CNT/PI (GCMCP) aerogel frame is schematically exhibited in Fig. 1a. Briefly, a homogeneous MXene/CNT/PAA ink is obtained by dispersing the MXene, CNT and PAA in deionized water with magnetic stirring. Then, MXene/CNT/PAA inks with different CNT contents are placed in the multiple feed system, and continuously deposited layer by layer on the plate through a 3D printer to prepare the gradient-conductive MXene/CNT/PAA gel frames. Afterward, with further freeze-drying for removing ice crystals and thermal imidization, a GCMCP aerogel frame with gradient-conductive and hierarchical porous structure is obtained. The successful thermal imidization of PAA into PI was verified by the FTIR spectra. As shown in Fig. S1a, new peaks located at 1720 cm^−1^ (C = O) and 1373 cm^−1^ (C-N) appear for MXene/CNT/PI, corresponding to the characteristic peaks of the imides in PI, indicating that the PAA is converted into PI [34, 35]. In addition, MXene exhibits excellent structural stability during thermal imidization (Fig. S1b). The average conductivity of MXene/CNT/PAA is 38.1 S cm^−1^, which slightly increases to 40 S cm^−1^ after thermal imidization. The increase in conductivity is attributed to the removal of inserted water and other molecules during imidization, thus reducing the interlayer spacing of MXene nanosheets. The strategy achieves the accurate construction and integrated molding of gradient-conductive structure and imparts hierarchical porous structure (lattice macrostructure and aerogel porous microstructure) to GCMCP aerogel frame (Fig. 1b). In the GCMCP aerogel frame, the conductivity gradually increases from top to bottom, where the top layer serving as the absorbing layer (impedance match layer) and the bottom layer as the high reflective layer (impedance mismatch layer), forming an absorption-reflection-reabsorption interface [36]. Moreover, the middle layer acts as the transition layer that can enable GCMCP aerogel frame to reflect and dissipate electromagnetic waves through multi-interface reflection. In addition, the lattice structure of GCMCP aerogel frame extends the transmission path of electromagnetic waves and increases the multiple reflections inside aerogel pore walls, which greatly improves the EMI shielding performance [37, 38]. The free electrons of the transition-metal carbide/nitride backbone endow MXene with metallic conductivity, which can dissipate electromagnetic waves through ohmic losses [39]. Furthermore, MXene nanosheets with Ti elements can form partial dipoles with surface functional groups (= O, −F, −OH) in electric fields and the strong electronegativity of F elements can induce polarization [40, 41]. The large mismatch of the interface between MXene and PI also leads to high interfacial polarization, conferring excellent absorption properties to the GCMCP aerogel frame [42, 43]. Therefore, the GCMCP aerogel frame with gradient-conductive structure can be further used to shield electromagnetic waves with low electromagnetic radiation pollution.

### Formation and Morphology of GCMCP Aerogel Frames

**Fig. 2:**
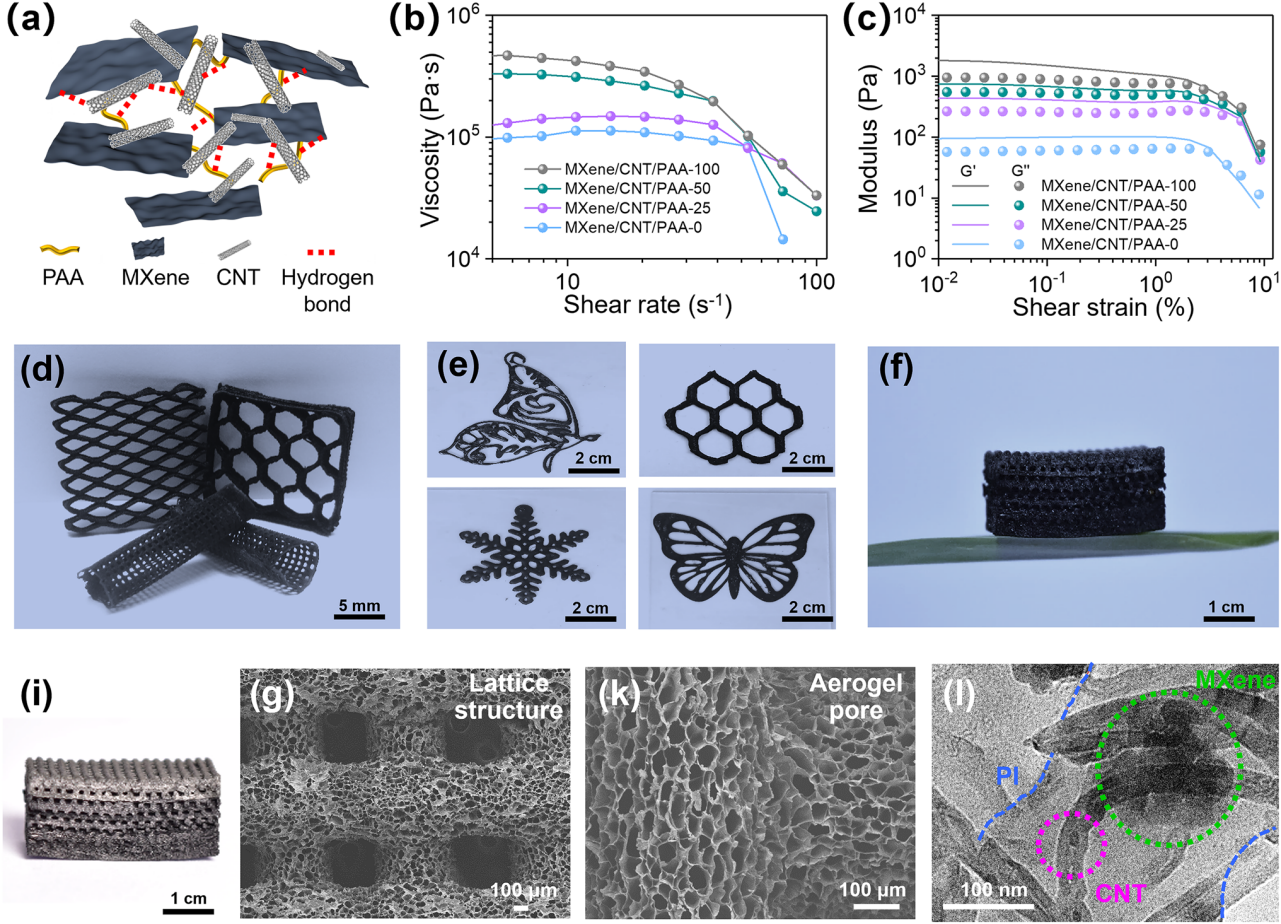
Formation and morphology of GCMCP aerogel frames. (**a**) Illustration of the interaction of MXene, CNT and PAA in MXene/CNT/PAA inks. (**b**) Log–log plots of viscosity versus shear rate of MXene/CNT/PAA inks. (**c**) Log–log plots of the storage modulus (G’) and loss modulus (G’’) versus shear strain of MXene/CNT/PAA inks. (**d–e**) Optical images demonstrating the GCMCP aerogel frames with different macrostructures by 3D printing. (**f**) Optical image demonstrating the lightweight of GCMCP aerogel frame. (**i**) Optical image of GCMCP aerogel frames and corresponding (**g–k**) SEM images. (**l**) TEM images of GCMCP aerogel showing the morphology of aerogel pore walls

The MXene/CNT/PAA inks with appropriate rheological properties are a prerequisite for 3D printing to accurately build gradient-conductive structure and hierarchical porous structure. The MXene sheets and CNT in the MXene/CNT/PAA inks serve as effective rheological modifiers by creating a stable structure through hydrogen bonding with PAA (Fig. 2a) [44, 45]. As shown by FTIR spectra in Fig. S2, the C = O peak of MXene/CNT/PAA shifts slightly from 1721 to 1719 cm^−1^, mainly due to the hydrogen bonding interaction between the C = O group in the PAA and the carboxyl/hydroxyl group in the MXene/CNT [34]. The two-dimensional MXene sheets with high aspect ratio and a monolithic layer structure are prepared by etching the Ti_3_AlC_2_ phase with LiF/HCl solution followed by exfoliation (Fig. S3a-b). Moreover, the successful preparation of MXene sheets is evidenced by the XRD patterns, that (104) peak almost disappears and (002) peak is broadened, implying the successful elimination of Al after etching and expansion of interlayer spacing (Fig. S3c) [46]. In addition, one-dimensional CNTs are used as bridges to connect two-dimensional MXene sheets, thus constructing a continuous conductive network [47]. The viscosity variations of MXene/CNT/PAA inks with different CNT contents (0, 25, 50, and 100 mg mL^−1^) are shown in Fig. 2b. The corresponding composite inks are named as MXene/CNT/PAA-0, MXene/CNT/PAA-25, MXene/CNT/PAA-50 and MXene/CNT/PAA-100, respectively (Table S1). All MXene/CNT/PAA inks show significant shear-thinning behaviors, which provides favorable conditions for continuous extrusion of inks. Furthermore, the storage modulus (G’) of all MXene/CNT/PAA inks is higher than the loss modulus (G’’) at low shear strain, showing the gel characteristics, which allows it self-supporting to maintain the printed structure (Fig. 2c) [45]. The stability of MXene/CNT/PAA inks with different CNT contents, further demonstrating good printability (Fig. S4). Therefore, the combination of continuous extrusion and self-supporting properties of MXene/CNT/PAA inks through 3D printing technology allows the integrated molding of high-precision GCMCP aerogel frames. As shown in Fig. 2d–e, GCMCP aerogel with various macroscopic structures, including honeycomb, diamond, cylindrical lattice structure, exquisite butterfly and snowflake, indicating the excellent printability of MXene/CNT/PAA inks. The printed GCMCP aerogel frame can stand on the bamboo leaves, indicating its lightweight nature (Fig. 2f).

The typical GCMCP aerogel frame with gradient-conductive structure and hierarchical porous structure (Fig. 2i). The color of the GCMCP aerogel frame gradually increased from top to bottom with the increase of CNT content. In addition, each layer is well connected, attributed to the strong hydrogen bonding effect of PAA in the MXene/CNT/PAA composite inks (Fig. S5). The SEM image of GCMCP exhibits a uniform lattice structure with filament spacing of 400 μm (Fig. 2g). Furthermore, the filament exhibits a typical aerogel structure with pore sizes ranging from 10 to 30 μm depending on various CNT contents (Figs. 2k and S6), resulting in a hierarchical porous structure consisting of lattice macrostructure and aerogel porous microstructure for the GCMCP aerogel frames. Moreover, MXene sheets and CNTs are interconnected and uniformly distributed in PI matrix, forming a continuous and strong conductive network in aerogel (TEM image in Fig. 2l). In addition, the energy-dispersive spectroscopy (EDS) mapping images also display the existence and distribution of carbon (C), nitrogen (N), oxygen (O), fluorine (F) and titanium (Ti) elements on the pore wall, further proving the uniform distribution of MXene and CNTs in PI matrix (Fig. S7). Moreover, the aerogel frame exhibits improved mechanical strength with the addition of CNT (Fig. S8), which provides the basis for constructing EMI shielding materials with good mechanical performance [29].

### EMI Shielding Performance of the MCP and GCMCP Aerogel Frames

**Fig. 3:**
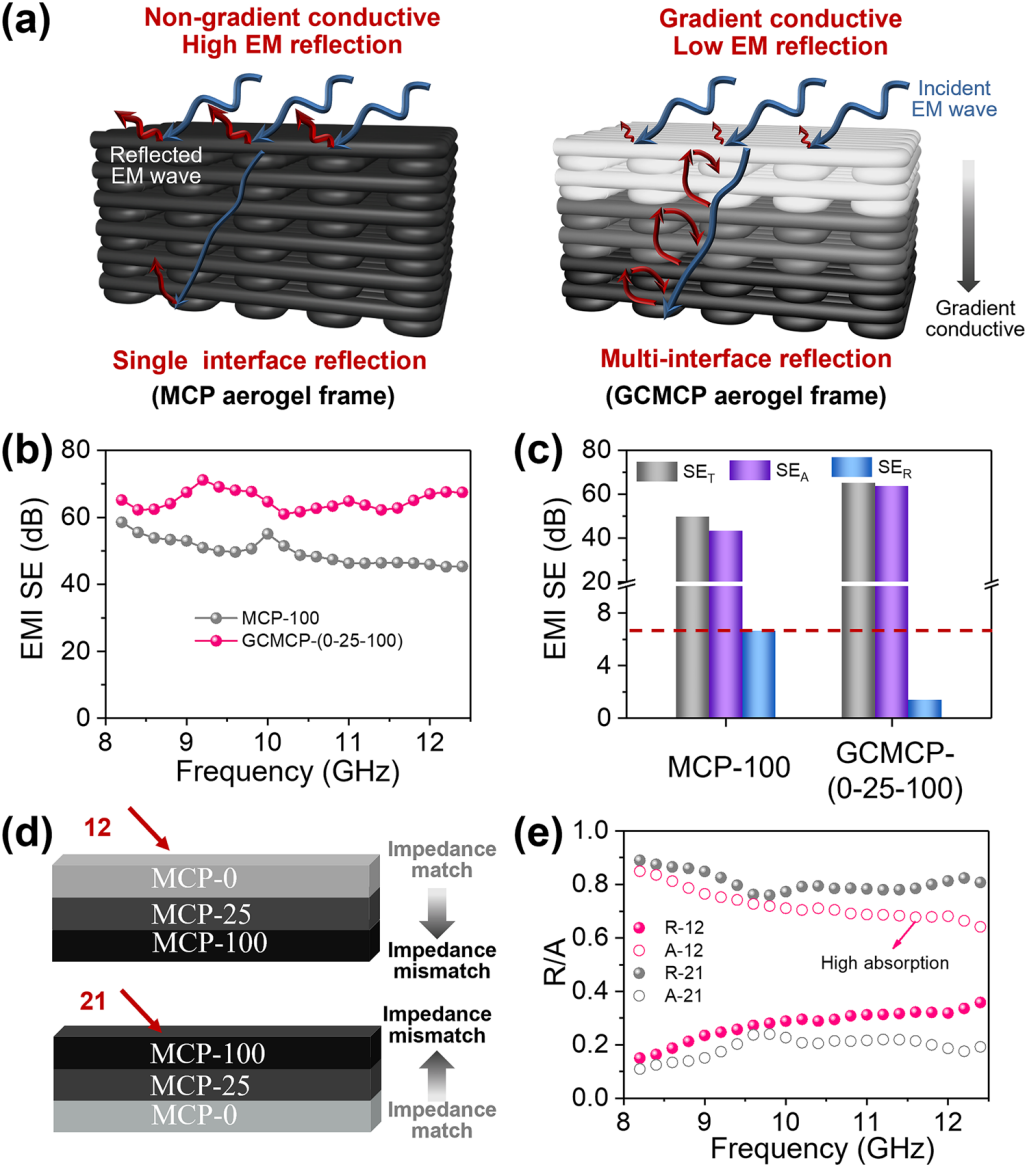
Electromagnetic shielding performance of GCMCP aerogel with gradient-conductive structure at X band (thickness: about 5 mm). (**a**) Schematic representation of shielding mechanism of non-gradient conductive MCP aerogel frame and gradient-conductive GCMCP aerogel frame. (**b**) EMI shielding performances and (**c**) SE_T_, SE_A_, and *SE*_R_ value of MCP-100 aerogel and GCMCP-(0–25-100) aerogel frame. (**d**) Schematic diagram of electromagnetic wave incident from different directions. (**e**) Reflection/absorption coefficient of GCMCP-(0–25-100) aerogel frame at different incident directions

To highlight the advantages of gradient-conductive structure in electromagnetic shielding, a non-gradient conductive MXene/CNT/PI-100 (MCP-100, 100 represents the content of CNT) aerogel frame is constructed for comparison (Fig. 3a). For the non-gradient conductive MCP-100 aerogel frame, the incident electromagnetic waves are easily reflected at the air-aerogel interface owing to high impedance mismatch, which inevitably brings the high secondary radiation pollution of electromagnetic waves. In contrast, the gradient-conductive GCMCP aerogel frame provides an impedance matching layer, so the incident wave can pass smoothly through the air-aerogel interface. And multi-interface reflections are generated between different conductive layers to continuously attenuate electromagnetic waves, which can reduce secondary radiation pollution of electromagnetic waves. In order to construct the gradient-conductive structure, the electrical conductivity and EMI shielding properties of each layer are adjusted by CNT contents. When the CNT contents of MCP-X (X = 0, 25, 50, 100; represents CNT contents) aerogel frame increase from 25 to 100 mg mL^−1^, the conductivity increases from 17.3 to 41.0 S m^−1^ and the EMI SE increases from 36.6 to 49 dB (Fig. S9a). Furthermore, MCP aerogel frames show a negligible decrease in conductivity after storing in 50 ℃ and 95% relative humidity environment for 2 days (Fig. S9b), indicating its good stability due to the protection of polyimide that can effectively prevent MXene from oxidation [35]. The GCMCP-(X_1_–X_2_–X_3_) aerogel frames with gradient-conductive structure are prepared by integrated and continuous 3D printing, in which X_1_, X_2_ X_3_ represent the CNT content in the top layer (impedance match layer), middle layer (transition layer) and bottom layer (impedance mismatch layer) (Table S2). The average EMI *SE* value at X band of GCMCP-(0–25–100) aerogel frame with gradient-conductive structure can reach 65.1 dB, which is higher than that of non-gradient conductive MCP-100 aerogel frame (EMI *SE* = 50 dB).

To further illustrate the EMI shielding mechanism of the GCMCP aerogel frames, the total EMI shielding effectiveness (*SE*_T_), shielding effectiveness of the reflection (*SE*_R_), and shielding effectiveness of the absorption (*SE*_A_) are shown in Fig. 3c. Compared with MCP-100 aerogel frame (*SE*_R_ = 6.6 dB), GCMCP-(0–25–100) aerogel frame has lower *SE*_R_ (1.4 dB), which can be attributed to that the impedance matching layer in GCMCP-(0–25–100) aerogel frame can reduce the reflection of electromagnetic waves on the air-aerogel interface. In addition, the high *SE*_A_ (63.7 dB) of GCMCP-(0–25–100) aerogel frame is attributed to the absorption-reflection-reabsorption interface in the gradient-conductive structure aerogel frame. Furthermore, the CNT content in each layer is adjusted to maximize the EMI shielding and electromagnetic wave adsorption. When the CNT content of bottom layer decreased to 50 mg mL^−1^, the conductivity of GCMCP-(0–25–50) aerogel frame decreases to 23.6 S m^−1^, and the corresponding EMI SE decreases to 47 dB (Figs. S10 and S11a), which is due to the low conductivity of bottom layer in the GCMCP-(0–25–50) aerogel frame resulting in weak electromagnetic losses. In addition, the conductivity of the GCMCP-(0–50–100) aerogel frame increased to 34.6 S cm^−1^ when the CNT content of the transition layer was increased to 50 mg mL^−1^. The corresponding EMI SE increased to 78.5 dB and the SE_R_ also increases to 7.5 dB (Fig. S11b). This high reflection owes to the large mismatch of conductivity in the interfaces between top layer and transition layer. Therefore, the reasonable gradient distribution of top layer (impedance match layer), middle layer (transition layer) and bottom layer (impedance mismatch layer) is of great significance to achieve high electromagnetic wave absorption and EMI shielding.

The EMI shielding performance of GCMCP aerogel frames with gradient-conductive structure is further investigated when electromagnetic waves incident from different incident directions. As shown in Fig. 3d–e, experiment “12” and experiment “21” represent the electromagnetic wave incident from the low conductive layer (MCP-0) and high conductive layer (MCP-100), respectively. When the electromagnetic waves are incident from the low conductive surface (MCP-0), the GCMCP-(0–25–100) aerogel frames have low average reflection coefficient (*R*, 0.27) and high average absorption coefficient (*A*, 0.73), which indicates that electromagnetic waves are dissipated by absorption (Figs. 3e and S12). In contrast, when the electromagnetic waves are incident from the high conductive surface (MCP-100), the GCMCP-(0–25–100) aerogel frames have high average *R* (0.81) and low average *A* (0.19), demonstrating that electromagnetic waves are almost reflected into free space due to the impedance mismatch of air-aerogel interfaces, causing secondary EM wave contamination. Therefore, the incident electromagnetic waves from the low conductive layer can benefit the high adsorption of electromagnetic waves, which highlights the merits of the unique asymmetric conductive structure.

### EMI Shielding Performance of GCMCP Aerogel Frames with Different Lattice Size

**Fig. 4:**
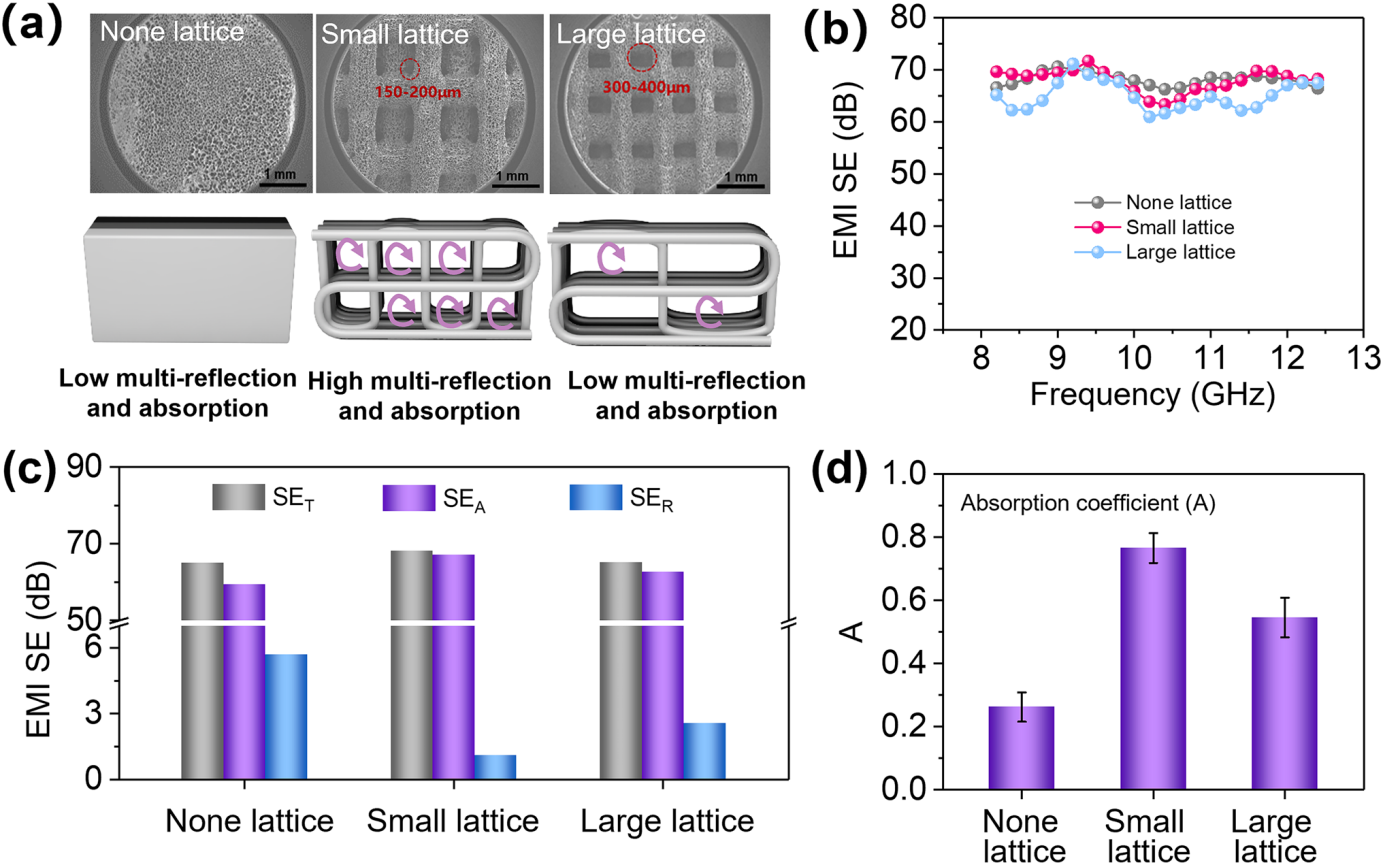
Electromagnetic shielding performance of GCMCP aerogel frames with different lattice size, thickness: about 5 mm. (**a**) SEM images and EMI shielding mechanism of GCMCP aerogel frames with different lattice size. (**b**) EMI *SE*, (**c**) *SE*_T_, *SE*_A_ and *SE*_R_ value and (**d**) absorption coefficient of none lattice, small lattice (150–200 μm) and large lattice (300–400 μm), respectively

Since the lattice structure of GCMCP aerogel frames could benefit to reduce the reflection and increase the absorption of electromagnetic waves, the effect of lattice size of aerogel frames on EMI shielding is further investigated. The design drawings and optical photographs of GCMCP aerogel frames with small and large lattice sizes are shown in Fig. S13. Figure 4a reveals the morphology and EMI shielding mechanism of GCMCP aerogel frames with different lattice size, where the small lattice size is 150–200 μm and the large lattice size is 300–400 μm. Compared with the non-lattice structure, the lattice structure introduced in GCMCP aerogel frame can lengthen the transmission path of electromagnetic waves inside the shielding material. At the same time, the hierarchical porous structure consisting of lattice structure and aerogel pores is more favorable to dissipate electromagnetic waves. It is worth noting that there is no significant change in EMI *SE* performance at X band as the lattice size increases (Fig. 4b). However, the SE_A_ is obviously improved for the small lattice structure (*SE*_A_ = 67 dB) as compared to the non-lattice (*SE*_A_ = 59.3 dB) and large lattice (*SE*_A_ = 62.6 dB) structure, realizing high absorption by printed structure optimization (Fig. 4c). Subsequently, we further tested the A value to evaluate the effect of lattice structure on electromagnetic waves absorption capacity, which is shown in Fig. 4d. It can be clearly observed that lattice structure with small size shows obvious higher A value of 0.77, indicating that the GCMCP aerogel frames with smaller lattice size have stronger ability to absorb electromagnetic waves. Therefore, GCMCP aerogel frame with gradient-conductive structure and hierarchical porous structure results in low reflection and high absorption, which effectively reduces electromagnetic wave reflection.

### Tunable EMI SE Performance of GCMCP Aerogel Frames with Different Thickness

**Fig. 5:**
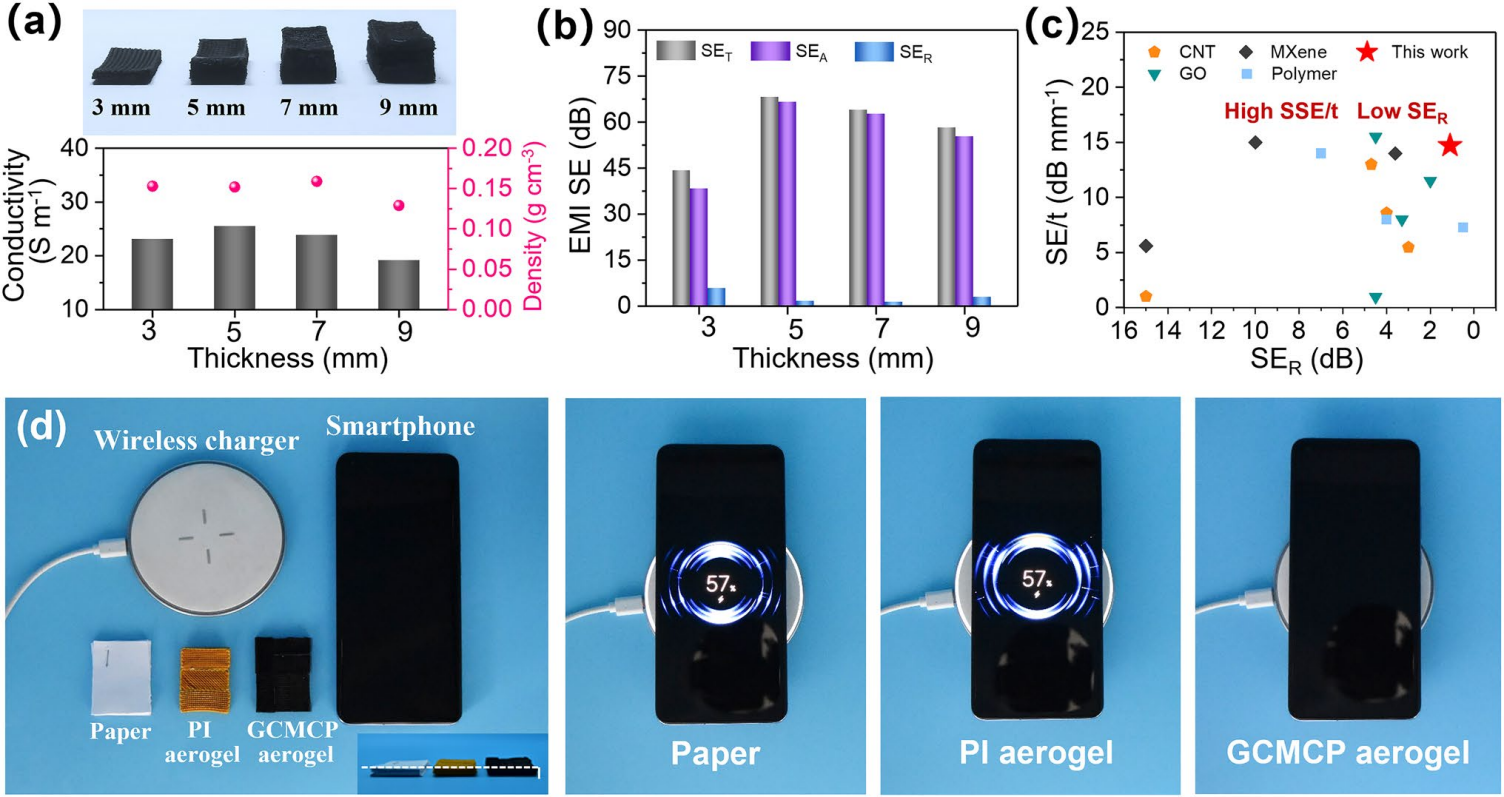
Electromagnetic shielding performance of GCMCP aerogel frame with various thicknesses. (**a**) Digital photographs of GCMCP aerogel frame with 3, 5, 7 and 9 mm, and the conductivity and density as a function of thickness. (**b**) *SE*_T_, *SE*_A_ and *SE*_R_ value of GCMCP aerogel frame with 3, 5, 7 and 9 mm, respectively. (**c**) Comparison of normalized EMI SE and SE_R_ with those of previously reported EMI shielding materials. (**d**) Demonstration of the EMI shielding ability of paper, PI aerogel frame and GCMCP aerogel frame for wireless charging

The normalized electromagnetic shielding effectiveness is an important criterion to evaluate the EMI shielding ability of porous materials, which includes three important parameters: the EMI *SE*, density and thickness. Here, we constructed GCMCP aerogel frames with thicknesses of 3, 5, 7 and 9 mm, respectively (Fig. 5a). The GCMCP aerogel frames have high conductivity (25.5 S m^−1^) and low density (0.152 g cm^−3^) at a thickness of 5 mm. Figure 5b displays the EMI *SE* performance of GCMCP aerogel frames with different thicknesses, and the GCMCP aerogel frame with thickness of 5 mm has the highest EMI *SE* of 68.2 dB and the lowest *SE*_R_ of 1.1 dB. However, the EMI *SE* of GCMCP aerogel frame decreases to 58.2 dB when the thickness increases to 9 mm. Theoretically, the impedance matching characteristic is usually measured by the Z_in_-1 value, which can be calculated by the following equation [48–50]:

8 $$ Z_{{{\text{in}}}} - 1 = \sqrt {\frac{\mu }{\varepsilon }} \tanh \left( {j2\pi \sqrt {\mu \varepsilon } f\frac{{\text{d}}}{c}} \right) - 1 $$

where $c$ and $f$ represent the light velocity (3 × 10^8^ m s^−1^) and EM frequency (8.2–12.4 GHz), $\varepsilon$ and $\mu$ represent the permittivity and permeability, and d indicates the thickness of aerogel frame. When the thickness of aerogel frame increases from 3 to 9 mm, the thickness of the impedance matching layer also increases from 1 to 3 mm, resulting in poor impedance matching and low EMI *SE*. We summarized the research progress of various EMI shielding materials in the literature, including MXene, CNT, polymer composite membranes/aerogels/frames [16, 51–62], and the results are shown in Fig. 5c and Table S3. The GCMCP aerogel frames with gradient-conductive structure and hierarchical porous structure exhibited high normalized EMI SE divided by thickness of 13.6 dB mm^−1^, and ultra-low *SE*_R_ of 1.1 dB, which is better than previously reported. In addition, benefiting from the low density (0.152 g cm^−3^) and high EMI shielding efficiency (68.2 dB) of GCMCP aerogel frames, the SSE (*SE*_T_/density) is up to 448.7 dB cm^3^ g^−1^.

As a proof of concept for evaluating the EMI shielding of GCMCP aerogel frames in practical conditions, we placed paper, PI aerogel frame and GCMCP aerogel frame with the same thickness between smartphone and wireless charger, to observe the charging situation of the smartphone (Fig. 5d and Movie S1). Apparently, the smartphone can be easily charged on wireless charger covered with paper and PI aerogel frame, but cannot be charged on covering with GCMCP aerogel frames, indicating that GCMCP aerogel frames can act as EMI shielding gasket and effectively block wireless power transmission process. Therefore, the 3D printing technology offers great flexibility for producing EMI shielding materials and devices with gradient-conductive structures for potential applications in electronic packaging.

## Conclusions

In summary, a gradient-conductive and hierarchical porous GCMCP aerogel frame is designed by 3D printing technology as a high-performance EMI shielding material with ultra-low reflection coefficient. The GCMCP aerogel frame with controllable gradient-conductivity are obtained by adjusting the CNT contents of MXene/CNT/PAA ink, based on the continuous “bottom-up” 3D printing technology with the multi-feeding system. In the GCMCP aerogel frames, the slightly conductive top layer serves as the EM absorption layer, while the highly conductive bottom layer as an EM reflection layer, forming an absorption-reflection-reabsorption interface. Moreover, the hierarchical porous structure in the GCMCP aerogel frame dramatically improved the electromagnetic wave entering the material for multiple reflection loss. These unique structure designs enable the GCMCP aerogel framework with high EMI *SE* (68.2 dB) and ultra-low *R* (0.23), compared with traditional polymer composite membranes/aerogels/frames. The integrated gradient-conductive GCMCP aerogel frames represent a promising future research direction for developing advanced EMI shielding materials for microelectronics.

### Supplementary Information

Below is the link to the electronic supplementary material.

Supplementary file1 (MP4 1179 KB)

Supplementary file2 (PDF 1035 KB)

## References

[1] Liu J, Zhang HB, Sun RH, Liu YF, Liu ZS (2017). Hydrophobic, flexible, and lightweight MXene foams for high-performance electromagnetic-interference shielding. Adv. Mater..

[2] Zong JY, Zhou XJ, Hu YF, Yang TB, Yan DX (2021). A wearable multifunctional fabric with excellent electromagnetic interference shielding and passive radiation heating performance. Compos. Part B Eng..

[3] Baan R, Grosse Y, Lauby-Secretan B, El Ghissassi F, Bouvard V (2011). Carcinogenicity of radiofrequency electromagnetic fields. Lancet Oncol..

[4] Liu XF, Li Y, Sun X, Tang WK, Deng G (2021). Off/on switchable smart electromagnetic interference shielding aerogel. Matter.

[5] Abbasi H, Antunes M, Velasco JI (2019). Recent advances in carbon-based polymer nanocomposites for electromagnetic interference shielding. Prog. Mater. Sci..

[6] Zhang YL, Gu JW (2022). A perspective for developing polymer-based electromagnetic interference shielding composites. Nano-Micro Lett..

[7] Yousefi N, Sun XY, Lin XY, Shen X, Jia JJ (2014). Highly aligned graphene/polymer nanocomposites with excellent dielectric properties for high-performance electromagnetic interference shielding. Adv. Mater..

[8] Yan DX, Pang H, Li B, Vajtai R, Xu L (2015). Structured reduced graphene oxide/polymer composites for ultra-efficient electromagnetic interference shielding. Adv. Funct. Mater..

[9] Cheng ML, Ying MF, Zhao RZ, Ji LZ, Li HX (2022). Transparent and flexible electromagnetic interference shielding materials by constructing sandwich AgNW@MXene/wood composites. ACS Nano.

[10] Wang M, Tang XH, Cai JH, Wu H, Shen JB (2021). Construction, mechanism and prospective of conductive polymer composites with multiple interfaces for electromagnetic interference shielding: A review. Carbon.

[11] Wang L, Ma ZL, Qiu H, Zhang YL, Yu Z (2022). Significantly enhanced electromagnetic interference shielding performances of epoxy nanocomposites with long-range aligned lamellar structures. Nano-Micro Lett..

[12] Guan QF, Han ZM, Yang KP, Yang HB, Ling ZC (2021). Sustainable double-network structural materials for electromagnetic shielding. Nano Lett..

[13] Chen YA, Potschke P, Pionteck J, Voit B, Qi HS (2020). Multifunctional cellulose/rGO/Fe_3_O_4_ composite aerogels for electromagnetic interference shielding. ACS Appl. Mater. Interfaces.

[14] Pan F, Rao YP, Batalu D, Cai L, Dong YY (2022). Macroscopic electromagnetic cooperative network-enhanced MXene/Ni chains aerogel-based microwave absorber with ultra-low matching thickness. Nano-Micro Lett..

[15] Zhao S, Zhang HB, Luo JQ, Wang QW, Xu B (2018). Highly electrically conductive three-dimensional Ti_3_C_2_T_x_ MXene/reduced graphene oxide hybrid aerogels with excellent electromagnetic interference shielding performances. ACS Nano.

[16] Zeng ZH, Wu TT, Han DX, Ren Q, Siqueira G (2020). Ultralight, flexible, and biomimetic nanocellulose/silver nanowire aerogels for electromagnetic interference shielding. ACS Nano.

[17] Xu YD, Yang YQ, Yan DX, Duan HJ, Zhao GZ (2018). Gradient structure design of flexible waterborne polyurethane conductive films for ultraefficient electromagnetic shielding with low reflection characteristic. ACS Appl. Mater. Interfaces.

[18] Hu YQ, Hou C, Shi YX, Wu JM, Yang D (2022). Freestanding Fe_3_O_4_/Ti_3_C_2_T_x_ MXene/polyurethane composite film with efficient electromagnetic shielding and ultra-stretchable performance. Nanotechnology.

[19] Xue B, Li Y, Cheng ZL, Yang SD, Xie L (2021). Directional electromagnetic interference shielding based on step-wise asymmetric conductive networks. Nano-Micro Lett..

[20] Lei ZM, Tian DK, Liu XB, Wei JH, Rajavel K (2021). Electrically conductive gradient structure design of thermoplastic polyurethane composite foams for efficient electromagnetic interference shielding and ultra-low microwave reflectivity. Chem. Eng. J..

[21] Yang JM, Liao X, Wang G, Chen J, Guo FM (2020). Gradient structure design of lightweight and flexible silicone rubber nanocomposite foam for efficient electromagnetic interference shielding. Chem. Eng. J..

[22] Bandyopadhyay A, Heer B (2018). Additive manufacturing of multi-material structures. Mater. Sci. Eng. R Rep..

[23] Müller LAE, Zimmermann T, Nyström G, Burgert I, Siqueira G (2020). Mechanical properties tailoring of 3D printed photoresponsive nanocellulose composites. Adv. Funct. Mater..

[24] Ambekar RS, Kushwaha B, Sharma P, Bosia F, Fraldi M (2021). Topologically engineered 3D printed architectures with superior mechanical strength. Mater. Today.

[25] Feng JZ, Su BL, Xia HS, Zhao SY, Gao C (2021). Printed aerogels: Chemistry, processing, and applications. Chem. Soc. Rev..

[26] Zong W, Chui NB, Tian ZH, Li YT, Yang C (2021). Ultrafine MOP nanoparticle splotched nitrogen-doped carbon nanosheets enabling high-performance 3D-printed potassium-ion hybrid capacitors. Adv. Sci..

[27] Kwon Y, Seo SE, Lee J, Berezvai S, Read de Alaniz J (2022). 3D-printed polymer foams maintain stiffness and energy dissipation under repeated loading. Compos. Commun..

[28] Pu L, Liu YP, Li L, Zhang C, Ma PM (2021). Polyimide nanofiber-reinforced Ti_3_C_2_T_x_ aerogel with "lamella-pillar" microporosity for high-performance piezoresistive strain sensing and electromagnetic wave absorption. ACS Appl. Mater. Interfaces.

[29] Lin CH, Luo SH, Meng FC, Xu B, Long T (2021). MXene/air-laid paper composite sensors for both tensile and torsional deformations detection. Compos. Commun..

[30] Xue TT, Zhu CY, Feng XL, Wali Q, Fan W (2022). Polyimide aerogel fibers with controllable porous microstructure for super-thermal insulation under extreme environments. Adv. Fiber Mater..

[31] Tian J, Yang Y, Xue TT, Chao GJ, Fan W (2022). Highly flexible and compressible polyimide/silica aerogels with integrated double network for thermal insulation and fire-retardancy. J. Mater. Sci. Technol..

[32] Zhu CY, Yang F, Xue TT, Wali Q, Fan W (2022). Metal-organic framework decorated polyimide nanofiber aerogels for efficient high-temperature particulate matter removal. Sep. Purif. Technol..

[33] Lu HC, Xia ZH, Zheng XJ, Mi QY, Zhang JM (2021). Patternable cellulose/MWCNT laminated nanocomposites with anisotropic thermal and electrical conductivity. Compos. Commun..

[34] Wang D, Peng YD, Dong JC, Pu L, Chang KQ (2023). Hierarchically porous polyimide aerogel fibers based on the confinement of Ti_3_C_2_T_x_ flakes for thermal insulation and fire retardancy. Compos. Commun..

[35] Liu J, Zhang HB, Xie X, Yang R, Liu Z (2018). Multifunctional, superelastic, and lightweight MXene/polyimide aerogels. Small.

[36] Ma L, Hamidinejad M, Zhao B, Liang CY, Park CB (2021). Layered foam/film polymer nanocomposites with highly efficient EMI shielding properties and ultralow reflection. Nano-Micro Lett..

[37] Xie ZX, Cai YF, Wei ZJ, Zhan YH, Meng YY (2022). Robust and self-healing polydimethylsiloxane/carbon nanotube foams for electromagnetic interference shielding and thermal insulation. Compos. Commun..

[38] Jiao ZB, Huyan WJ, Yang F, Yao JR, Tan RY (2022). Achieving ultra-wideband and elevated temperature electromagnetic wave absorption via constructing lightweight porous rigid structure. Nano-Micro Lett..

[39] Li QY, Liu ML, Zhong BC, Zhang WQ, Jia ZX (2022). Tetramethylammonium hydroxide modified MXene as a functional nanofiller for electrical and thermal conductive rubber composites. Compos. Commun..

[40] Zhou TZ, Yu YZ, He B, Wang Z, Xiong T (2022). Ultra-compact MXene fibers by continuous and controllable synergy of interfacial interactions and thermal drawing-induced stresses. Nat. Commun..

[41] Shahzad F, Alhabeb M, Hatter CB, Anasori B, Man Hong S (2016). Electromagnetic interference shielding with 2D transition metal carbides (MXenes). Science.

[42] Zeng ZH, Wu N, Wei JJ, Yang YF, Wu TT (2022). Porous and ultra-flexible crosslinked MXene/polyimide composites for multifunctional electromagnetic interference shielding. Nano-Micro Lett..

[43] Yang XQ, Zhang YF, Luo JM, Tusiime R, Lu CZ (2022). Fe_3_O_4_ uniformly decorated reduced graphene oxide aerogel for epoxy nanocomposites with high emi shielding performance. Compos. Commun..

[44] Liu H, Chen XY, Zheng YJ, Zhang DB, Zhao Y (2021). Lightweight, superelastic, and hydrophobic polyimide nanofiber /MXene composite aerogel for wearable piezoresistive sensor and oil/water separation applications. Adv. Funct. Mater..

[45] Yang Y, Fan W, Yuan SJ, Tian J, Chao GJ (2021). A 3D-printed integrated MXene-based evaporator with a vertical array structure for salt-resistant solar desalination. J. Mater. Chem. A.

[46] Liu JJ, Yang WJ, Xu Y, Yuen ACY, Chen TBY (2022). MXene-based films via scalable fabrication with improved mechanical and antioxidant properties for electromagnetic interference shielding. Compos. Commun..

[47] Zhu TY, Feng QC, Wan KN, Zhang C, Li B (2022). Articular cartilage-inspired 3D superelastic and fatigue-resistant spongy conductors against harsh environments. Sci. China Mater..

[48] Deng ZM, Tang PP, Wu XY, Zhang HB, Yu ZZ (2021). Superelastic, ultralight, and conductive Ti_3_C_2_T_x_ MXene/acidified carbon nanotube anisotropic aerogels for electromagnetic interference shielding. ACS Appl. Mater. Interfaces.

[49] Li XL, Yin XW, Xu HL, Han MK, Li MH (2018). Ultralight mxene-coated, interconnected sicnws three-dimensional lamellar foams for efficient microwave absorption in the x-band. ACS Appl. Mater. Interfaces.

[50] Cole KS, Cole RH (1941). Dispersion and absorption in dielectrics i alternating current characteristics. J. Chem. Phys..

[51] Huangfu YM, Ruan KP, Qiu H, Lu YJ, Liang CB (2019). Fabrication and investigation on the PANi/MWCNT/thermally annealed graphene aerogel/epoxy electromagnetic interference shielding nanocomposites. Compos. Part A Appl. Sci. Manuf..

[52] Zeng ZH, Jin H, Chen MJ, Li WW, Zhou LC (2016). Lightweight and anisotropic porous MWCNT/WPU composites for ultrahigh performance electromagnetic interference shielding. Adv. Funct. Mater..

[53] Pei XY, Liu GD, Shao RQ, Yu RR, Chen RX (2022). 3D-printing carbon nanotubes/Ti3C2Tx/chitosan composites with different arrangement structures based on ball milling for emi shielding. J. Appl. Polym. Sci..

[54] Shen B, Li Y, Zhai WT, Zheng WG (2016). Compressible graphene-coated polymer foams with ultralow density for adjustable electromagnetic interference (EMI) shielding. ACS Appl. Mater. Interfaces.

[55] Wan YJ, Zhu PL, Yu SH, Sun R, Wong CP (2017). Ultralight, super-elastic and volume-preserving cellulose fiber/graphene aerogel for high-performance electromagnetic interference shielding. Carbon.

[56] Fan ZM, Wang DL, Yuan Y, Wang YS, Cheng ZJ (2020). A lightweight and conductive MXene/graphene hybrid foam for superior electromagnetic interference shielding. Chem. Eng. J..

[57] Shen B, Zhai WT, Tao MM, Ling JQ, Zheng WG (2013). Lightweight, multifunctional polyetherimide/graphene@Fe_3_O_4_ composite foams for shielding of electromagnetic pollution. ACS Appl. Mater. Interfaces.

[58] Wang YY, Sun WJ, Yan DX, Dai K, Li ZM (2021). Ultralight carbon nanotube/graphene/polyimide foam with heterogeneous interfaces for efficient electromagnetic interference shielding and electromagnetic wave absorption. Carbon.

[59] Yu Z, Dai TW, Yuan SW, Zou HW, Liu PB (2020). Electromagnetic interference shielding performance of anisotropic polyimide/graphene composite aerogels. ACS Appl. Mater. Interfaces.

[60] Li XH, Li XF, Liao KN, Min P, Liu T (2016). Thermally annealed anisotropic graphene aerogels and their electrically conductive epoxy composites with excellent electromagnetic interference shielding efficiencies. ACS Appl. Mater. Interfaces.

[61] Zeng ZH, Wang CX, Zhang YF, Wang PY, Seyed Shahabadi SI (2018). Ultralight and highly elastic graphene/lignin-derived carbon nanocomposite aerogels with ultrahigh electromagnetic interference shielding performance. ACS Appl. Mater. Interfaces.

[62] Xia BH, Zhang XH, Jiang J, Wang Y, Li T (2022). Facile preparation of high strength, lightweight and thermal insulation polyetherimide/Ti_3_C_2_T_x_ MXenes/Ag nanoparticles composite foams for electromagnetic interference shielding. Compos. Commun..

