# Supplementary material for: 3D Printed Integrated Gradient-Conductive MXene/CNT/Polyimide Aerogel Frames for Electromagnetic Interference Shielding with Ultra-Low Reflection
Source: Nanomicro Lett. 2023 Feb 8;15:45. doi: 10.1007/s40820-023-01017-5 (PMC9908813; doi:10.1007/s40820-023-01017-5)
Supplement: Supplementary file 2 — Supplementary file2 (PDF 1035 KB) [file 40820_2023_1017_MOESM2_ESM.pdf]

Supporting Information for

3D Printed Integrated Gradient-Conductive MXene/CNT/Polyimide Aerogel Frames for Electromagnetic Interference Shielding with Ultra-Low Reflection

Tiantian Xue[1], Yi Yang[1], Dingyi Yu[1], Qamar Wali[4], Zhenyu Wang[3], Xuesong Cao[3], Wei Fan[1, 2, *], Tianxi Liu[1, 2, *]

[1] State Key Laboratory for Modification of Chemical Fibers and Polymer Materials, College of Materials Science and Engineering, Donghua University, 2999 North Renmin Road, Shanghai 201620, P. R. China

[2] Key Laboratory of Synthetic and Biological Colloids, Ministry of Education, School of Chemical and Material Engineering, Jiangnan University, Wuxi 214122, P. R. China

[3] Institute of Environmental Processes and Pollution control, School of Environment and Civil Engineering, Jiangnan University, Wuxi 214122, P. R. China

[4] NUTECH School of Applied Sciences & Humanities, National University of Technology, Islamabad 44000, Pakistan

* Corresponding authors. E-mail: weifan@dhu.edu.cn (Wei Fan); txliu@jiangnan.edu.cn (Tianxi Liu)

Supplementary Figures and Tables

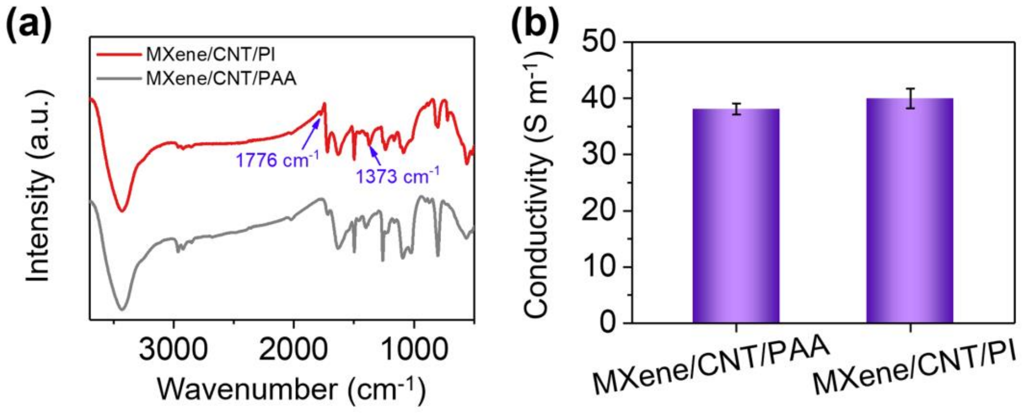

Fig. S1 a FTIR spectra of the MXene/CNT/PAA and MXene/CNT/PI. **b** Conductivity of the MXene/CNT/PAA and MXene/CNT/PI

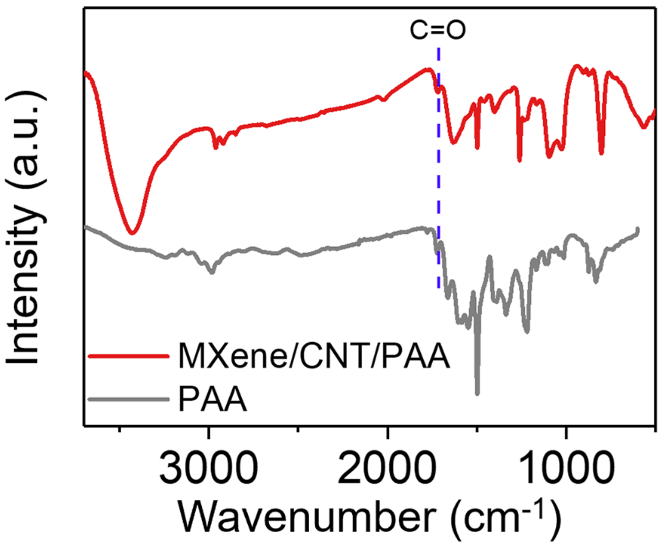

Fig. S2 FTIR spectra of the PAA and MXene/CNT/PAA

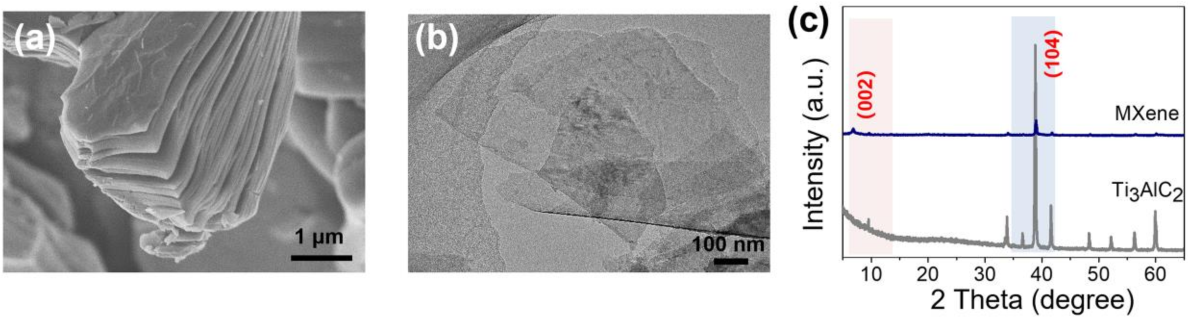

Fig. S3 a SEM image of the multi-layered MXene. **b** TEM image of the few-layered MXene. **c** The XRD spectrum of the MAX(Ti_3AlC_2) and MXene

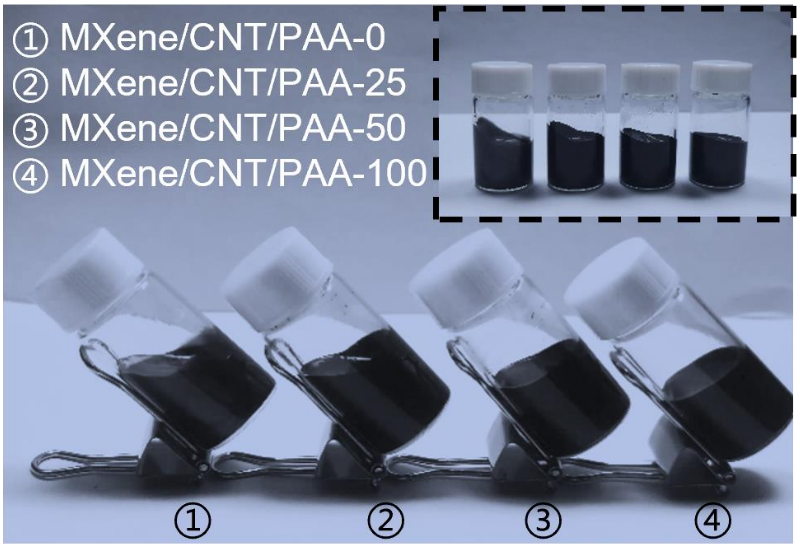

Fig. S4 Optical picture of MXene/CNT/PAA composite inks with different CNT contents

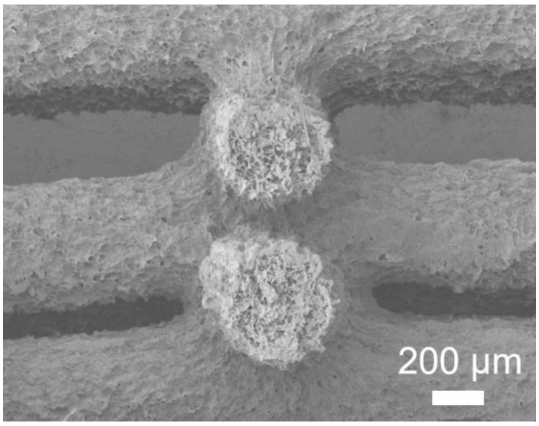

Fig. S5 Cross-sectional SEM images of GCMCP aerogel frames

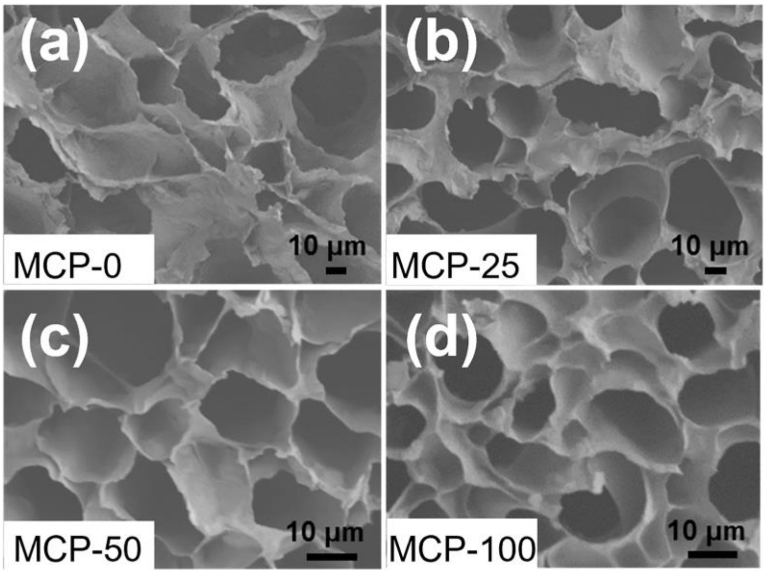

Fig. S6 a-b SEM images of MCP aerogel with various CNT contents

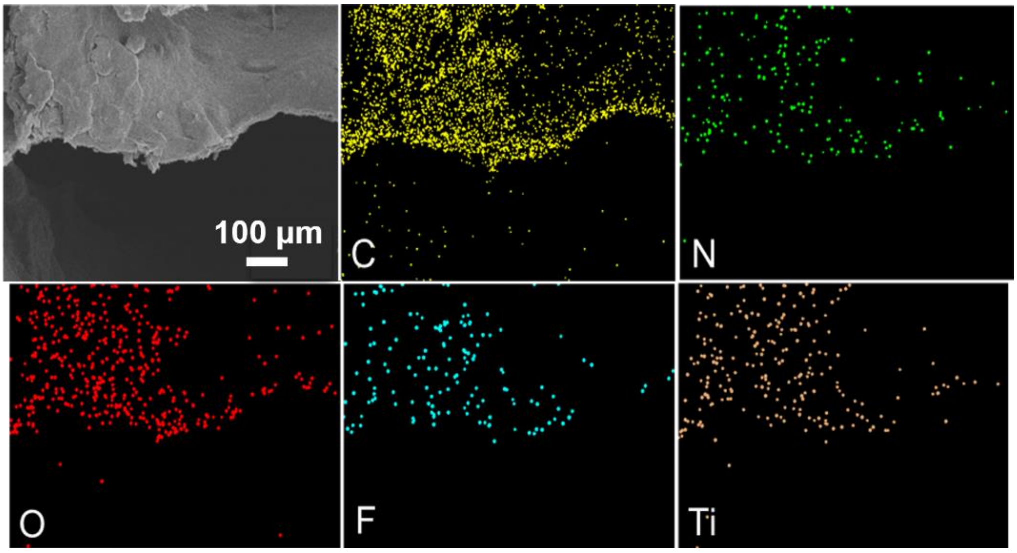

Fig. S7 SEM image of GCMCP aerogel wall and EDS mapping images of C, N, O, F, and Ti elements

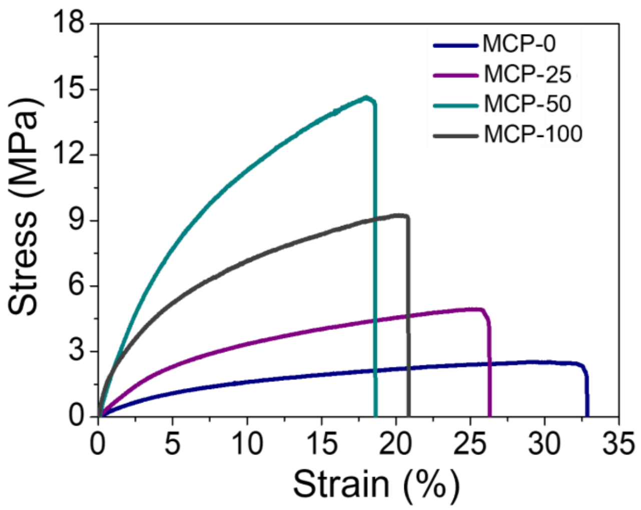

Fig. S8 The strain-stress curves of MCP aerogel as a function of CNT contents

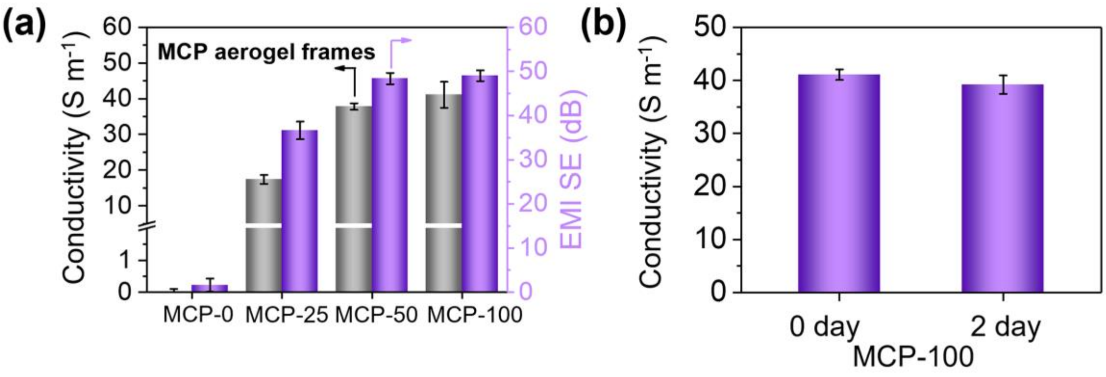

Fig. S9 a Conductivity and EMI SE value of MCP aerogel as a function of CNT contents. **b** Conductivity of the MCP-100 aerogel frame after stored in a 95% RH environment and a temperature of 50 °C for different days

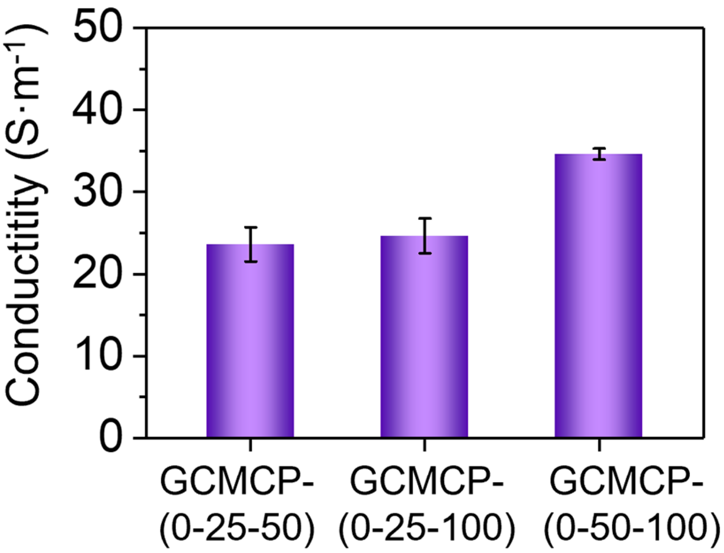

Fig. S10 Conductivity of GCMCP aerogel frame

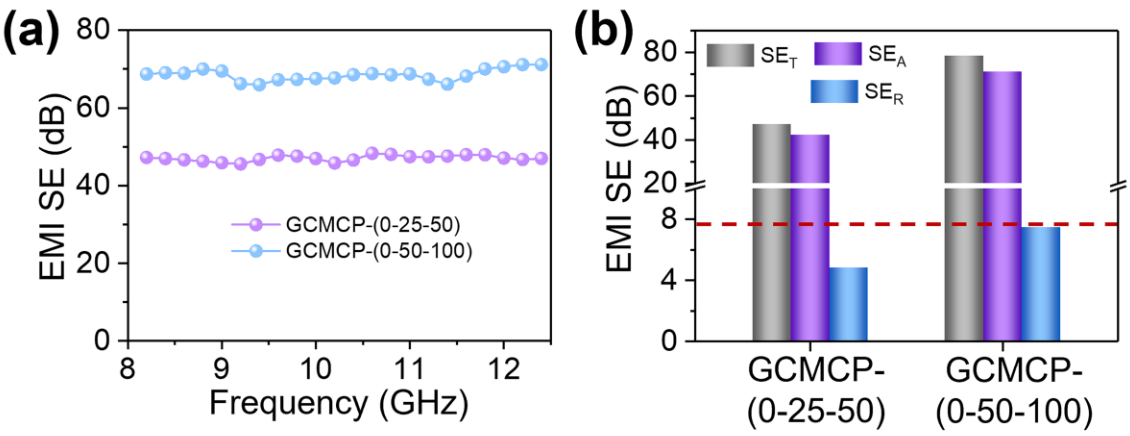

Fig. S11 a EMI shielding performances of GCMCP aerogel frame. **b** The SE_T, SE_A, SE_R value of GCMCP aerogel frame

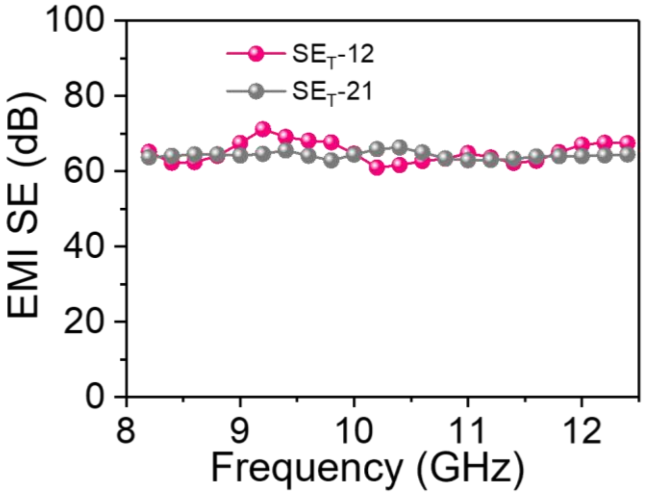

Fig. S12 EMI shielding performances of GCMCP- (0-25-100) aerogel frame at different incident directions

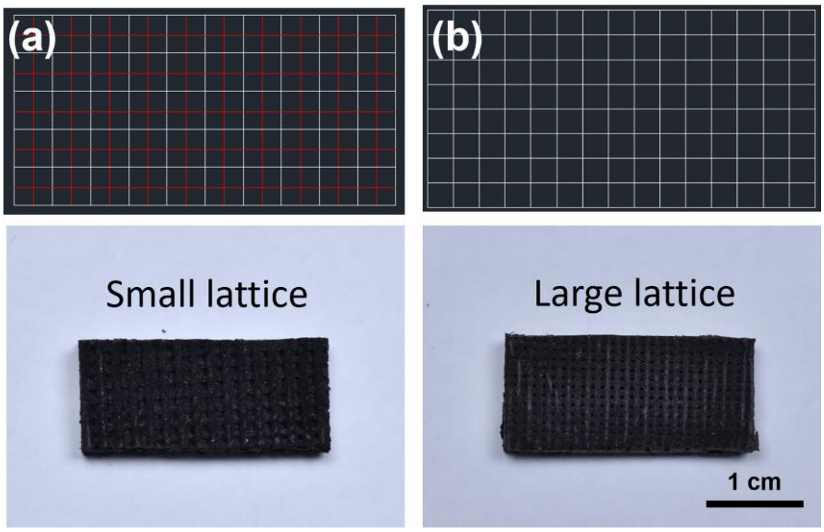

Fig. S13 a-b Digital images of GCMCP aerogel frames with different lattice size

Table S1 Composition of MXene/CNT/PAA composite inks

No.	MXene:PAA	CNT (mg mL^{-1})	Inks	Aerogel
1	1:1	0	MXene/CNT/PAA-0	MCP-0
2	1:1	25	MXene/CNT/PAA-25	MCP-25
3	1:1	50	MXene/CNT/PAA-50	MCP-50
4	1:1	100	MXene/CNT/PAA-100	MCP-100

Table S2 Composition of the GCMCP aerogel frames with a thickness of 9 mm

No.	Top layer (3 mm)	Middle layer (3 mm)	Bottom layer (3 mm)	Name
1	MCP-0	MCP-25	MCP-50	MCP-(0-25-50)
2	MCP-0	MCP-25	MCP-100	MCP-(0-25-100)
3	MCP-0	MCP-50	MCP-100	MCP-(0-50-100)

Table S3 The electromagnetic shielding performance of the representative literature

Materials	Thickness (mm)	EMI SE (dB)	SE_R (dB)	SE/t (dB mm^{-1})	SSE (dB cm^3 g^{-1})	Refs.
3D printed GO/CNT/PLA material	4.29	36.8	4	8.58	/	[S1]
MWCNT/WPU aerogel	4.5	50	15	1	1148	[S2]
3D printed MXene/CNT/chitosan aerogel	2	26	4.7	13	1944	[S3]
Carbon nanotube/graphene/polyimide foam	5	28.2	3	5.64	16890	[S4]
Polyimide/graphene aerogel	2.5	28.8	2	11.5	343	[S5]

Graphene/polyurethane foam	60	57.7	4.5	0.96	458	[S6]
Graphene aerogel	4	32	3.3	8	/	[S7]
Graphene/lignin-derived carbon aerogel	2	30.9	4.5	15.5	4955.6	[S8]
Polyetherimide/MXene/Ag nanoparticle foam	2	28	3.6	14	/	[S9]
rGO/MXene aerogel	8.9	50	15	5.6	6217	[S10]
AgNWs/PDMS aerogel	4	62	10	15.5	23888	[S11]
PANI/MWCNT/thermally annealed graphene aerogel/epoxy	3	42	7	14	/	[S12]
G@Fe3O4/PEI aerogel	2.5	18.2	0.5	7.28	41.5	[S13]
GF@PDMS aerogel	4.5	36.1	4	8	16890	[S14]
GCMCP aerogel	5	68.2	1.1	13.6	448.7	This work

Movie S1: GCMCP aerogel frame as electromagnetic shielding gasket can effectively prevent the wireless charging process of smartphone

Supplementary References

[S1] S.H. Shi, Z. Peng, Z.l. Peng, J.J. Jing, L. Yang et al., 3D printing of delicately controllable cellular nanocomposites based on polylactic acid incorporating graphene/carbon nanotube hybrids for efficient electromagnetic interference shielding. ACS Sustainable Chem. Eng. **8**, 7962-7972 (2020). https://doi.org/10.1021/acssuschemeng.0c01877

[S2] Z.H. Zeng, H. Jin, M.J. Chen, W.W. Li, L.C. Zhou, Z. Zhang, Lightweight and anisotropic porous MWCNT/WPU composites for ultrahigh performance electromagnetic interference shielding. Adv. Funct. Mater. **26**(2), 303-310 (2016). https://doi.org/10.1002/adfm.201503579

[S3] X.Y. Pei, G.D. Liu, R.Q. Shao, R.R. Yu, R.X. Chen et al., 3D-printing carbon nanotubes/$Ti_3C_2T_x$/chitosan composites with different arrangement structures based on ball milling for EMI shielding. J. Appl. Polym. Sci. **139**(45), e53125 (2022) https://doi.org/10.1002/app.53125

[S4] Y.J. Wan, P.L. Zhu, S.H. Yu, R. Sun, C.P. Wong et al., Ultralight, super-elastic and volume-preserving cellulose fiber/graphene aerogel for high-performance electromagnetic interference shielding. Carbon **115**, 629-639 (2017). https://doi.org/10.1016/j.carbon.2017.01.054

[S5] Z. Yu, T.W. Dai, S.W. Yuan, H.W. Zou, P.B. Liu, Electromagnetic interference shielding performance of anisotropic polyimide/graphene composite aerogels. ACS Appl. Mater. Interfaces **12**(27), 30990-31001 (2020). https://doi.org/10.1021/acsami.0c07122

[S6] B. Shen, Y. Li, W.T. Zhai, W.G. Zheng, Compressible graphene-coated polymer foams with ultralow density for adjustable electromagnetic interference (EMI) shielding. ACS Appl. Mater. Interfaces **8**(12), 8050-8057 (2016). https://doi.org/10.1021/acsami.5b11715

[S7] X.H. Li, X.F. Li, K.N. Liao, P. Min, T. Liu et al., Thermally annealed anisotropic graphene aerogels and their electrically conductive epoxy composites with excellent electromagnetic interference shielding efficiencies. ACS Appl. Mater. Interfaces **8**(48), 33230-33239 (2016). https://doi.org/10.1021/acsami.6b12295

[S8] Z.H. Zeng, C.X. Wang, Y.F. Zhang, P.Y. Wang, S.I. Seyed Shahabadi et al., Ultralight and highly elastic graphene/lignin-derived carbon nanocomposite aerogels with ultrahigh electromagnetic interference shielding performance. ACS Appl. Mater. Interfaces **10**(9), 8205-8213 (2018). https://doi.org/10.1021/acsami.7b19427

[S9] B.H. Xia, X.H. Zhang, J. Jiang, Y. Wang, T. Li et al., Facile preparation of high strength, lightweight and thermal insulation polyetherimide/$Ti_3C_2T_x$ MXenes/Ag nanoparticles composite foams for electromagnetic interference shielding. Compos. Commun. **29**, 101028 (2022). https://doi.org/10.1016/j.coco.2021.101028

[S10] Z.M. Fan, D.L. Wang, Y. Yuan, Y.S. Wang, Z.J. Cheng et al., A lightweight and conductive MXene/graphene hybrid foam for superior electromagnetic interference shielding. Chem. Eng. J. **381**, 122696 (2020). https://doi.org/10.1016/j.cej.2019.122696

[S11] Z.H. Zeng, T.T. Wu, D.X. Han, Q. Ren, G. Siqueira et al., Ultralight, flexible, and biomimetic nanocellulose/silver nanowire aerogels for electromagnetic interference shielding. ACS Nano **14**(3), 2927-2938 (2020). https://doi.org/10.1021/acsnano.9b07452

[S12] Y.M. Huangfu, K.P. Ruan, H. Qiu, Y.J. Lu, C.B. Liang et al., Fabrication and investigation on the PANi/MWCNT/thermally annealed graphene aerogel/epoxy electromagnetic interference shielding nanocomposites. Compos. Part A Appl. Sci. Manuf. **121**, 265-272 (2019). https://doi.org/10.1016/j.compositesa.2019.03.041

[S13] B. Shen, W.T. Zhai, M.M. Tao, J.Q. Ling, W.G. Zheng, Lightweight, multifunctional polyetherimide/graphene@Fe_3O_4 composite foams for shielding of electromagnetic pollution. ACS Appl. Mater. Interfaces **5**(21), 11383-11391

(2013). https://doi.org/10.1021/am4036527

[S14] Y.J. Wan, P.L. Zhu, S.H. Yu, R. Sun, C.P. Wong et al., Ultralight, super-elastic and volume-preserving cellulose fiber/graphene aerogel for high-performance electromagnetic interference shielding. Carbon **115**, 629-639 (2017). https://doi.org/10.1016/j.carbon.2017.01.054
